# Impaired large-scale cortico–hippocampal network connectivity, including the anterior temporal and posterior medial systems, and its associations with cognition in patients with first-episode schizophrenia

**DOI:** 10.3389/fnins.2023.1167942

**Published:** 2023-06-05

**Authors:** Kangkang Xue, Jingli Chen, Yarui Wei, Yuan Chen, Shaoqiang Han, Caihong Wang, Yong Zhang, Xueqin Song, Jingliang Cheng

**Affiliations:** ^1^Department of Magnetic Resonance Imaging, The First Affiliated Hospital of Zhengzhou University, Zhengzhou, China; ^2^Key Laboratory for Functional Magnetic Resonance Imaging and Molecular Imaging of Henan Province, Zhengzhou, China; ^3^Engineering Technology Research Center for Detection and Application of Brain Function of Henan Province, Zhengzhou, China; ^4^Engineering Research Center of Medical Imaging Intelligent Diagnosis and Treatment of Henan Province, Zhengzhou, China; ^5^Key Laboratory of Magnetic Resonance and Brain Function of Henan Province, Zhengzhou, China; ^6^Key Laboratory of Brain Function and Cognitive Magnetic Resonance Imaging of Zhengzhou, Zhengzhou, China; ^7^Key Laboratory of Imaging Intelligence Research Medicine of Henan Province, Zhengzhou, China; ^8^Engineering Research Center of Brain Function Development and Application of Henan Province, Zhengzhou, China; ^9^Department of Psychiatry, The First Affiliated Hospital of Zhengzhou University, Zhengzhou, China

**Keywords:** schizophrenia, functional connectivity, magnetic resonance imaging, cognition, hippocampus

## Abstract

**Background and objective:**

The cortico–hippocampal network is an emerging neural framework with striking evidence that it supports cognition in humans, especially memory; this network includes the anterior temporal (AT) system, the posterior medial (PM) system, the anterior hippocampus (aHIPPO), and the posterior hippocampus (pHIPPO). This study aimed to detect aberrant patterns of functional connectivity within and between large-scale cortico–hippocampal networks in first-episode schizophrenia patients compared with a healthy control group via resting-state functional magnetic resonance imaging (rs-fMRI) and to explore the correlations of these aberrant patterns with cognition.

**Methods:**

A total of 86 first-episode, drug-naïve schizophrenia patients and 102 healthy controls (HC) were recruited to undergo rs-fMRI examinations and clinical evaluations. We conducted large-scale edge-based network analysis to characterize the functional architecture of the cortico–hippocampus network and investigate between-group differences in within/between-network functional connectivity. Additionally, we explored the associations of functional connectivity (FC) abnormalities with clinical characteristics, including scores on the Positive and Negative Syndrome Scale (PANSS) and cognitive scores.

**Results:**

Compared with the HC group, schizophrenia patients exhibited widespread alterations to within-network FC of the cortico–hippocampal network, with decreases in FC involving the precuneus (PREC), amygdala (AMYG), parahippocampal cortex (PHC), orbitofrontal cortex (OFC), perirhinal cortex (PRC), retrosplenial cortex (RSC), posterior cingulate cortex (PCC), angular gyrus (ANG), aHIPPO, and pHIPPO. Schizophrenia patients also showed abnormalities in large-scale between-network FC of the cortico–hippocampal network, in the form of significantly decreased FC between the AT and the PM, the AT and the aHIPPO, the PM and the aHIPPO, and the aHIPPO and the pHIPPO. A number of these signatures of aberrant FC were correlated with PANSS score (positive, negative, and total score) and with scores on cognitive test battery items, including attention/vigilance (AV), working memory (WM), verbal learning and memory (Verb_Lrng), visual learning and memory (Vis_Lrng), reasoning and problem-solving (RPS), and social cognition (SC).

**Conclusion:**

Schizophrenia patients show distinct patterns of functional integration and separation both within and between large-scale cortico–hippocampal networks, reflecting a network imbalance of the hippocampal long axis with the AT and PM systems, which regulate cognitive domains (mainly Vis_Lrng, Verb_Lrng, WM, and RPS), and particularly involving alterations to FC of the AT system and the aHIPPO. These findings provide new insights into the neurofunctional markers of schizophrenia.

## 1. Introduction

Schizophrenia is a form of serious mental disorder with complicated causes, a prolonged course, and a high recurrence rate, and it can potentially lead to terminal mental disability. The clinical symptoms of schizophrenia are extremely intractable, including hallucination, delusion, speech and behavior disorder, apathy, social withdrawal, and cognitive impairment (Andreasen, 1999). Cognitive impairment is one of the main clinical signs of schizophrenia, bringing about a long-term detrimental effect on the social functioning of patients. Consistent deficits across a multitude of cognitive domains, including basic cognition (such as perception, attention, memory, spatial function, and executive function) and advanced cognition (such as emotional cognition, attribution style, abstract thinking ability, and language), have been reported in schizophrenia patients in previous studies; these deficits are considered to be the endophenotype of schizophrenia. A meta-analysis of cognitive function in schizophrenia has found that memory may be the most severely impaired cognitive function (Aleman et al., 1999), including episodic memory and working memory. The accuracy of memory in schizophrenia patients is significantly reduced by the impact of mental symptoms and emotional disorders (Tan et al., 2021), which are characterized by universality and non-selectivity and occur in approximately 50% of schizophrenia patients, but the neurobiological source of these impairments remains unclear.

The medial temporal lobe (MTL), which encompasses the hippocampus and adjacent cortex areas such as the perirhinal cortex (PRC) and parahippocampal cortex (PHC), has long been thought to represent the neurological underpinning of cognition, especially memory (Eichenbaum et al., 2007), and this can be reflected in alterations to cortical and subcortical activities and in the functional connectivity (FC) of large-scale brain networks (Rugg and Vilberg, 2013; Ross et al., 2018). However, an accumulation of evidence shows that distinct regions of the MTL exhibit diverse characteristics, supporting the presence of various modules of cognition and memory (Ranganath, 2010). As a pivot, the hippocampus communicates with intra-MTL sub-cortical regions and extra-MTL cortical regions, and there is also intimate connectivity among various cortical regions. Considering the separation between the anterior and posterior MTL (Ranganath and Ritchey, 2012), Ritchey et al. (2015) proposed a framework including the anterior temporal (AT) system and posterior medial (PM) system, namely, the “PMAT” framework. The AT system, with the perirhinal cortex (PRC) as a core component, includes the inferior temporal cortex (ITC), orbitofrontal cortex (OFC), fusiform gyrus (FUS), and amygdala (AMYG), and is related to project information management and long-term retention of learning items in the form of concepts. In contrast, the PM system, with the parahippocampal cortex (PHC) and retrosplenial cortex (RSC) as its core components, includes the angular gyrus (ANG), posterior cingulate cortex (PCC), and precuneus (PREC), and participates in contextual information management and long-term retention of learning items in the form of scenario schemas (Cooper and Ritchey, 2019). Moreover, the anterior and posterior hippocampus are distinctively integrated into the AT and PM systems, which contribute to the respective cognitive processes, including object memory, scene memory, and spatial memory, considering multiple scales of representation along the hippocampal anteroposterior axis (Poppenk et al., 2013; Strange et al., 2014; Collin et al., 2015; Berron et al., 2018; Brunec et al., 2018). Although Berron et al. have found that the medial temporal lobe in patients with Alzheimer's disease shows decreased functional connectivity with the AT and PM systems and is closely related to cognition and memory impairment (Berron et al., 2020), it is not clear how the anterior and posterior hippocampus function in relation to the PM and AT, respectively, and which functional connectivity is responsible for specific cognition domains. There is also no conclusion on how the PMAT framework mediates cognitive dysfunction in schizophrenia patients, especially memory impairment, or whether it mediates the positive and negative symptoms of schizophrenia through aberrant intra- and inter-network functional connectivity. Therefore, the AT system, the PM system, and the anterior and posterior hippocampus can together be considered as a cortico–hippocampal network that mediates cognitive disorders. Distinct brain regions within the entire network are considered independent units, with close connectivity between them. In addition, each subnetwork can also be considered as a new, larger unit, which may interlink with other subnetworks.

The functional magnetic resonance imaging (fMRI) technique can be applied to reliably identify the neurobiological underpinnings of cognition deficits in schizophrenia, with linked functional brain regions exhibiting increased or decreased activity in a resting state; nevertheless, the heterogeneity of brain regions in various studies undoubtedly makes this approach challenging. Resting-state fMRI is a relatively novel method that is considered to have more advantages than task-based research, especially in the study of schizophrenia, because it is easy to perform, does not involve any complex tasks, and may overcome the limitations of task-induced fMRI research. Numerous studies have discovered that schizophrenia patients exhibit aberrant brain activity across a variety of paradigms associated with the unique symptom domains of schizophrenia. With regard to the cognition domain, researchers have set about associating the internal neural signals acquired by resting-state fMRI (rs-fMRI) with cognitive behavioral phenotypes; this approach is believed to offer a richer characterization of cognitive impairment in clinical populations such as schizophrenia patients. However, it is laborious to identify specific patterns of neurological impairment for diverse cognitive-domain defects, which has naturally become a thorn in the side for researchers, who expect to recognize specific neurobiological issues by identifying the associations between particular cognitive-domain hurdles and fMRI aberrations. Based on this foundation, numerous researchers have discovered that the activities of certain brain regions are significantly linked to a variety of cognitive domains. Scholars have found that executive functioning is strongly related to activities in the dorsolateral prefrontal cortex (dlPFC) and its connectivity with subcortical and cerebellar regions (Cole et al., 2011; Repovs et al., 2011; Su et al., 2013; Tu et al., 2013; Wang et al., 2014). Repovs et al. (2011) and Unschuld et al. (2014) discovered that dysfunction of the prefrontal cortex, and especially frontal-parietal connectivity, affects impairment of working memory. Several researchers have reported on the correlation between processing speed, attention, and the interconnectivity of the default mode network (DMN), despite inconsistent results (Bassett et al., 2012; He et al., 2013; Moran et al., 2013; Mwansisya et al., 2013; Argyelan et al., 2014). The neurobiological mechanism of situational memory is poorly understood, although Camchong et al. (2011) observed using the Wechsler Memory Scale that the decreased situational memory of schizophrenia is related to the functional connectivity between the medial frontal lobe and the entire brain. Employing the method of graph theory, Lynall et al. (2010) investigated the correlation between verbal knowledge and the loss of overall integration, as well as the absence of tight connections in the entire brain network. Integrating the information summarized above, the specific brain network corresponding to the cognitive deficits occurring in schizophrenia and the ways in which diverse target brain regions mediate cognitive impairment through connectivity alteration are still poorly understood, despite the fact that the associated neurobiological research on cognition in schizophrenia has made significant strides.

Against the backdrop of the description of human brain networks via fMRI, researchers have gradually become keen to explore the modes of intra- and inter-network connectivity for networks composed of various modules and to investigate whether impairments may occur to the balance between functional integration and separation of brain network modules (Bertolero et al., 2015; Sporns and Betzel, 2016). Although several studies have presented evidence that schizophrenia patients show impairments to large-scale brain network connectivity, their findings have not been completely clear on the module integration and separation of cognition-related networks in schizophrenia. Despite findings that the functional connectivity of the bilateral hippocampus with several brain areas related to episodic memory (such as the posterior cingulate cortex, extrastriate cortex, medial prefrontal cortex, and parahippocampal gyrus) is reduced in schizophrenia (Zhou et al., 2008), and structural MRI studies that have found that the destruction of cortico–hippocampal anatomical connectivity is strongly associated with schizophrenia (Qiu et al., 2010), there is no research on the connectivity between the hippocampal subregion and related cortical networks in the anterior and posterior MTL, or their correlation with cognition. At present, there has been no study on the PMAT framework in schizophrenia patients, although several studies have found that the functional connectivity of the MTL is related to memory encoding in schizophrenia (Haut et al., 2015), which indirectly reflects the possible correlation between schizophrenia and structural and functional alterations of the PMAT framework. An increase or decrease of FC between different brain regions within the cortico–hippocampal network in schizophrenia patients, established via fMRI, can be taken as a reflection of the integrated activity of the network and thereby its relevance; this is known as “within-network FC analysis.” In addition, detecting an increase or decrease in FC between different subnetworks (the AT system, PM system, anterior hippocampus, and posterior hippocampus) within the cortico–hippocampal network may reflect the integration and separation of distinct modules within this network and the mediation of different cognitive domains; this is known as “between-network FC analysis.” Between-network FC of the AT, PM, aHIPPO, and pHIPPO may reveal the stability and synergy of the cortico–hippocampal network, and stronger between-network FC of the AT and PM would imply that these regions are more integrated. In addition, differences in FC between the aHIPPO, pHIPPO, AT, and PM systems, as well as correlations with distinct cognitive domains, can reveal the functional separation of the subnetworks. We expect that individuals with schizophrenia may show poorer network collaboration compared to normal individuals, which may manifest as decreased FC between networks.

In this study, we employed the resting-state fMRI method to characterize FC alterations in the cortico–hippocampal network in 86 first-episode schizophrenia patients and 102 healthy controls. We calculated edge-based FC in a large-scale network and examined the integration and separation of cortico–hippocampal network modules, including the AT system, the PM system, and anterior and posterior hippocampus. To assess the clinical relevance of observed functional alterations, we correlated these with PANSS scores and scores on cognitive test battery items in schizophrenia. We hypothesized that FC within and between cortico–hippocampal subsystems in schizophrenia patients would alter and would thus be correlated with cognition, especially memory; such associations could assist us in better comprehending the mechanism underlying cognitive decline in early schizophrenia.

## 2. Materials and methods

### 2.1. Participants

We recruited 86 first-episode drug-naïve schizophrenia patients and 102 healthy controls; all participants were of Han Chinese ethnicity and right-handed. In accordance with the Diagnostic and Statistical Manual of Mental Disorders, Fourth Edition (DSM-IV), schizophrenia was diagnosed by two professional clinical psychiatrists. The patients included in this study had never received treatment or psychological counseling, and their duration of disease was <3 years. Two trained researchers assessed the patients using questionnaires and assessed their symptoms on the Positive and Negative Syndrome Scale (PANSS). The inclusion criteria for the participants were as follows: (1) age 13–60 years old, (2) education to primary school level or above, and (3) Han Chinese ethnicity and right-handed.

The cognition assessment of schizophrenia patients was conducted by a professionally trained psychiatrist using the Chinese version of the MATRICS Consensus Cognitive Battery (MCCB). The subtests of the MCCB include: (1) the Trail Making Test (TMT); (2) a symbol-coding test; (3) the Hopkins Verbal Learning Test (HVLT); (4) a spatial span test; (5) the Mazes test; (6) the Brief Visuospatial Memory Test (BVMT) of visual learning; (7) a category fluency test; (8) the Mayer–Salovey–Caruso Emotional Intelligence Test (MSCEIT); and (9) the Continuous Performance Test—Identical Pairs (CTP-IP). The cognitive test results were transformed using cognitive statistics software into scores on seven domains of cognition and then statistically analyzed. Specifically, these seven domains were speed of processing (SOP), attention/vigilance (AV), working memory (WM), verbal learning and memory (Vrbl_Lrng), visual learning and memory (Vis_Lrng), reasoning and problem-solving (RPS), and social cognition (SC).

The present study was approved by the Ethics Committee of the First Affiliated Hospital of Zhengzhou University. The exclusion criteria for this study were as follows: (1) head trauma or severe organic brain disease; (2) drug or alcohol abuse; (3) organic mental disorder; (4) pregnancy; and (5) MRI contraindication. Additional exclusion criteria for the HC group were diseases of the nervous system, mental illness, and family history of mental illness. After the study had been explained, all participants were asked to sign an informed consent sheet.

### 2.2. Data acquisition

Magnetic resonance imaging (MRI) data were acquired using a 3.0 T MRI scanner (Discovery MR750, GE, USA) with an 8-channel head coil. All subjects were asked to lie quietly with their eyes closed, and to remain awake and relaxed; foam pads and rubber earplugs were used to reduce head movement and interference from noise.

A 3D-T1 BRAVO sequence was applied to obtain high-resolution structural images with the following parameters: repetition time (TR)/echo time (TE) = 8.2/3.2 ms, slices = 188, slice thickness = 1 mm, slice gap = 0 mm, flip angle (FA) = 12°, field of view (FOV) = 25.6 × 25.6 cm^2^, number of averages = 1, data matrix = 256 × 256, voxel size = 1 × 1 × 1 mm^3^, and scan time = 4.33 min. The functional images were obtained using a gradient spin echo-planar imaging (EPI) sequence: TR/TE = 2,000/30 ms, slices = 32, slice thickness = 4 mm, slice gap = 0.5 mm, FA = 90°, FOV = 22 × 22 cm^2^, number of averages = 1, data matrix = 64 × 64, voxel size = 3.4375 × 3.4375 × 4 mm^3^, and 180 volumes lasting for 360 s.

### 2.3. Data preprocessing

Using the MATLAB (MathWorks) platform, the DPABI toolbox (http://rfmri.org/dpabi, V6.1_220101) was used to preprocess rs-fMRI data. To allow for magnetic saturation, the first five volumes of data were disregarded. Next, additional preprocessing was conducted via the following procedures: (1) slice timing; (2) realignment, in which subjects with maximum head motion exceeding 3 mm or 3° rotation were removed for the purpose of head motion rectification; (3) normalization (DARTEL method), in which data on segmentation of the unified structural images were used to register individual functional images to the Montreal Neurological Institute (MNI) coordinate space, resampling to 3 × 3 × 3 mm^3^; (4) detrending; (5) temporal band-pass filtering (0.01–0.08 Hz); and (6) regression for nuisance covariates of the white matter, cerebrospinal fluid, and head movement profile (Friston-24).

### 2.4. Large-scale network calculation

Data were processed using DPABI (Yan et al., 2016), following the methods published by Li et al. (2021). The DPABINet software package was used to process network calculations. We defined a total of 24 anatomical regions of interest (ROIs) according to the Harvard–Oxford atlas in order to extract the average BOLD signal of all voxels in each ROI. Additionally, hippocampal masks were manually adjusted, and regions of interest were separated in the anterior and posterior sections at the uncal apex (foci at or anterior to y = 21 mm in MNI space may be regarded as falling within the anterior hippocampus, as this coordinate incorporates the uncal apex in the MNI152 template and current neuroanatomical atlases) (Berron et al., 2020). First, we extracted the average time series from each ROI and calculated the Pearson correlation coefficient for each ROI, which is taken as a measure of functional connectivity (FC); subsequently, Fisher's r-to-z transformation was applied to all FC values, forming a final 24 × 24 FC matrix.

To investigate the interconnections between each large-scale network, the 24 ROIs were further divided into four subsystems, namely, the AT (PRC, ITC, OFC, FUS, and AMYG), the PM (PHC, RSC, ANG, PCC, and PREC), the anterior hippocampus (Ahippo), and the posterior hippocampus (Phippo) systems. We took the average FC of all ROIs in each subsystem network and the average FC z-score of all connectivity edges between subsystems to represent the FC between subsystems, normalized by the product of the number of nodes within each of the four subsystems (Gu et al., 2015).

### 2.5. Statistical analysis

Statistical Package for the Social Sciences (SPSS) version 26.0 was employed for the statistical processing of general demographic and clinical data, which were represented in the form of mean ± standard deviation. Age and education level were compared between the SCH group and the HC group using two-sample *t*-tests, and gender representation was compared using the chi-square test. Cognitive scores were compared between the SCH group and the HC group using two-sample *t*-tests. In all cases, statistical significance was determined by a threshold of *p* < 0.05.

The fMRI data were statistically analyzed using the DPABINet software package. Two-sample *t*-tests were used to compare the FC of every ROI between the SCH and HC groups, with age, gender, years of education, and head motion as covariates (voxels threshold of *p* < 0.001, cluster threshold of *p* < 0.05, false discovery rate correction, two-tailed).

Statistically significant values for FC at the ROI level and subnetwork level were generated individually. Subsequently, using the SPSS software package, Spearman's correlation analysis was conducted to explore the correlation of extracted FC values with cognitive scores and PANSS scores, with Bonferroni correction and with age, gender, years of education, and head motion as covariates (*p* < 0.05).

## 3. Results

### 3.1. Clinical and demographic characteristics

There were no significant differences in age (*t* = 0.446, *p* = 0.656), gender (χ^2^ = 0.418, *p* = 0.518), education level (*t* = 0.923, *p* = 0.357), or head motion (*t* = 1.437, *p* = 0.152) between the SCH and HC groups (Table 1). PANSS scores for the SCH group and cognitive scores for the SCH group and HC groups are shown in Table 2: the SCH group presented with lower cognitive scores on SOP, AV, WM, Verb_Lrng, Vis_Lrng, RPS, and SC relative to the HC group (Table 2).

**Table 1:** Demographic data for the SCH and HC groups.

**Demographic variable**	**SCH (*n =* 86)** **(Mean ±SD)**	**HC (*n =* 102)** **(Mean ±SD)**	***t/χ^2^***	***P***
Age (years)	21.87 ± 8.36	21.39 ± 6.95	0.45	0.656
Sex (male/female)	44/42	57/45	0.42	0.518
Education (years)	10.68 ± 2.71	11.04 ± 2.90	0.92	0.357
Mean FD (mm)	0.060 ± 0.044	0.068 ± 0.035	1.44	0.152

The sex difference between the SCH and HC groups was examined via the chi-square test. Differences in continuous variables between the SCH and HC groups were examined via two-sample *t*-tests. SCH, schizophrenia patients; HC, healthy control; FD, framewise displacement; SD, standard deviation.

**Table 2:** Clinical data for the SCH and HC groups.

**Clinical variable**	**SCH (*n =* 86) (Mean ±SD)**	**HC (*n =* 102) (Mean ±SD)**	***t***	***P***
**PANSS**				
Positive	20.02 ± 6.54			
Negative	21.25 ± 7.23			
General	41.13 ± 11.02			
Total score	82.39 ± 21.71			
**MCCB**				
SOP	26.85 ± 12.58	40.53 ± 10.01	−7.50	0.000
AV	29.76 ± 12.68	46.42 ± 11.24	−8.70	0.000
WM	38.22 ± 11.92	44.1 ± 9.74	−3.22	0.002
Verb_Lrng	36.55 ± 8.932	42.12 ± 8.09	−4.10	0.000
Vis_Lrng	36.02 ± 16.35	42.41 ± 9.45	−3.07	0.003
RPS	33.12 ± 9.47	36.74 ± 7.56	−2.63	0.009
SC	36.33 ± 15.60	40.53 ± 7.47	−2.22	0.028

Differences in cognitive scores on the MCCB between the SCH and HC groups were examined via two-sample *t*-tests. SCH, schizophrenia group; HC, healthy control group; PANSS, Positive and Negative Syndrome Scale; MCCB, MATRICS Consensus Cognitive Battery; SOP, speed of processing; AV, attention/vigilance; WM, working memory; Vrbl_Lrng, verbal learning and memory; Vis_Lrng, visual learning and memory; RPS, reasoning and problem-solving; SC, social cognition.

### 3.2. Large-scale network FC

#### 3.2.1. Group differences in FC within networks

**Figure 1:**
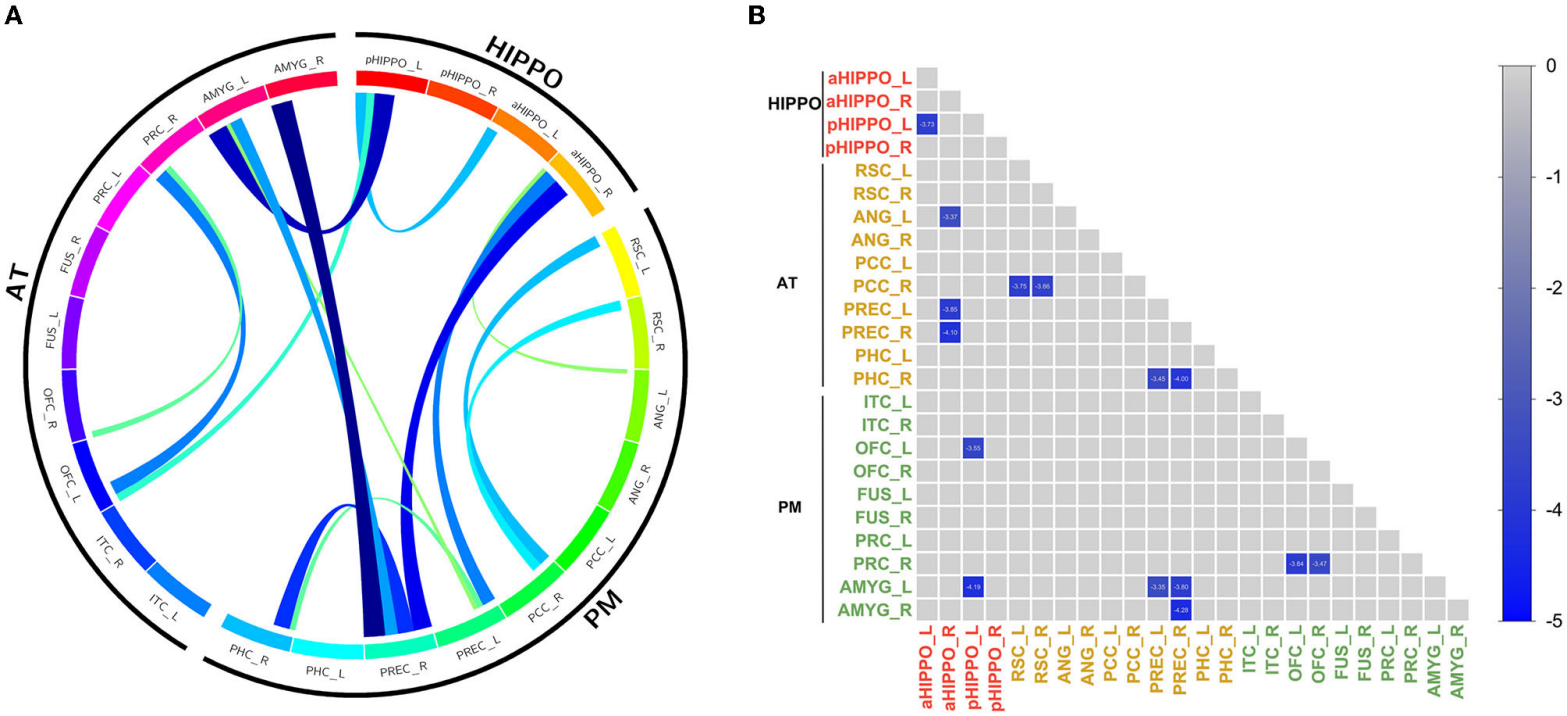
Results of within-network functional connectivity analyses. **(A)** Differences in within-network functional connectivity between the SCH and HC groups. **(B)** Within-network functional connectivity matrix. Pairwise correlations within the cortico–hippocampal network were averaged across subjects. Cooler colors represent decreased within-network FC in schizophrenia patients compared to healthy controls. PREC, precuneus; AMYG, amygdala; pHIPPO, posterior hippocampus; aHIPPO, anterior hippocampus; PHC, parahippocampal cortex; OFC, orbitofrontal cortex; PRC, perirhinal cortex; RSC, retrosplenial cortex; PCC, posterior cingulate cortex; ANG, angular gyrus; ITC, inferior temporal cortex; FUS, fusiform gyrus; L, left; R, right; PM, posterior medial system; AT, anterior temporal system; HIPPO, hippocampus.

**Figure 2:**
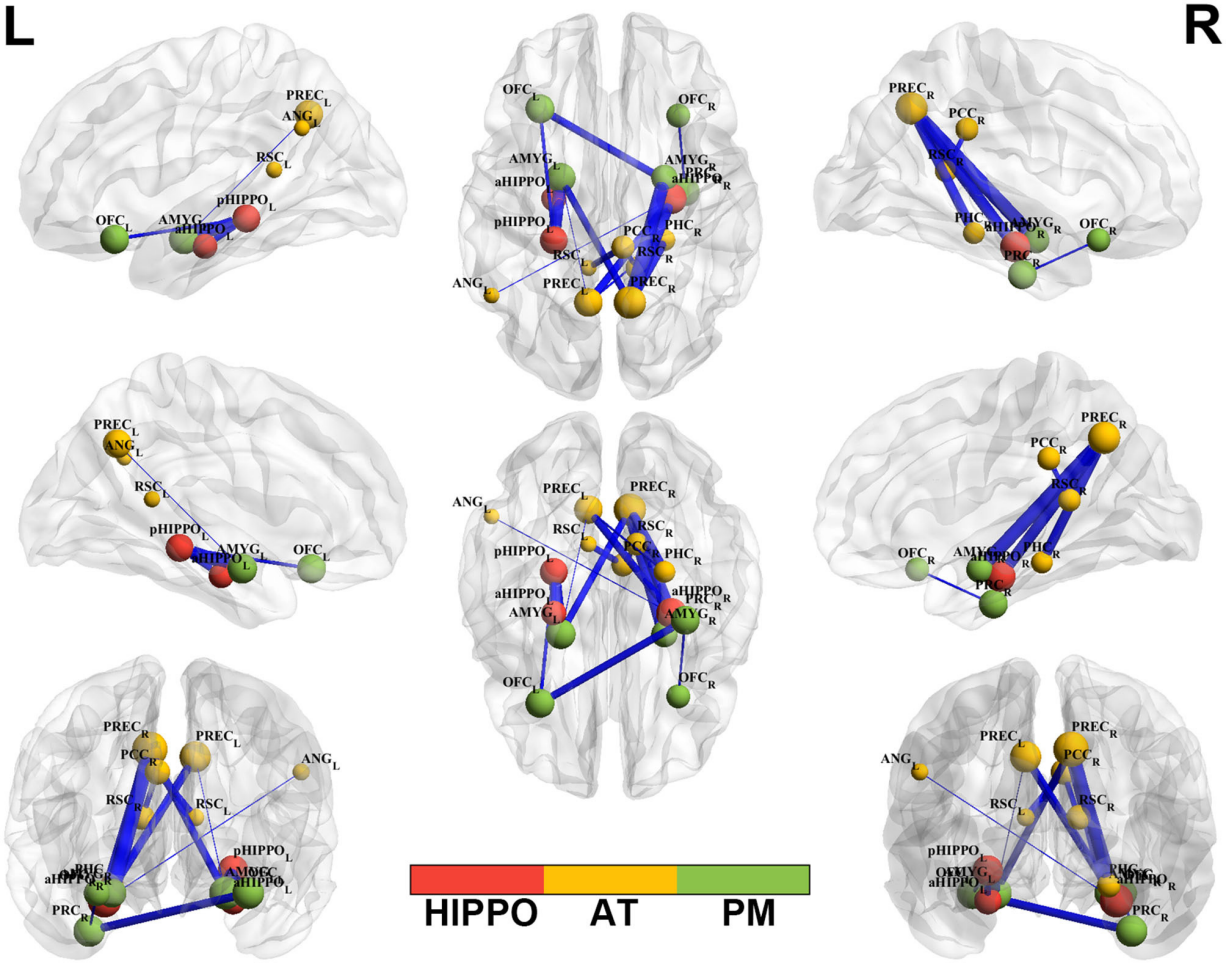
Altered edge-based functional connectivity between the SCH and HC groups. Brain maps show the affected edges (lines) and their connecting nodes (spheres) from several perspectives. The size of a sphere indicates how many affected edges are connected to this node: bigger nodes have more affected edges than smaller ones. The color of a node indicates which network it belongs to: red represents the HIPPO, yellow represents the AT, and green represents the PM. The cold color of the edges indicates decreased FC. Cooler blue edges represent stronger reductions in FC among the SCH group compared to the HC group. L, left; R, right; PM, posterior medial system; AT, anterior temporal system; HIPPO, hippocampus.

Compared to the HC group, SCH patients showed 15 significantly different edges within the cortico–hippocampal network, representing significantly decreased FC between the right PCC and the left RSC; the right PCC and the right RSC; the left aHIPPO and the left pHIPPO; the left OFC and the left pHIPPO; the left AMYG and the left pHIPPO; the left ANG and the right aHIPPO; the left PREC and the right aHIPPO; the right PREC and the right aHIPPO; the right PHC and the left PREC; the left AMYG and the left PREC; the right PHC and the right PREC; the left AMYG and the right PREC; the right AMYG and the right PREC; the right PRC and the left OFC; and the right PRC and the right OFC (Figure 1, Table 3). These sites of decreased FC were involved in the AT system, the PM system, the aHIPPO, and the pHIPPO (Figure 2).

**Table 3:** Differences in large-scale within-network FC.

**ROI-wise FC (SCH > HC)**	***t***	***P***
PREC (R)–AMYG (R)	−4.28	0.000
pHIPPO (L)–AMYG (L)	−4.19	0.000
aHIPPO (R)–PREC (R)	−4.10	0.000
PREC (R)–PHC (R)	−4.00	0.000
aHIPPO (R)–PREC (L)	−3.85	0.000
OFC (L)–PRC (R)	−3.84	0.000
PREC (R)–AMYG (L)	−3.80	0.000
RSC (L)–PCC (R)	−3.75	0.000
aHIPPO (L)–pHIPPO (L)	−3.73	0.000
RSC (R)–PCC (R)	−3.66	0.000
pHIPPO (L)–OFC (L)	−3.55	0.000
OFC (R)–PRC (R)	−3.47	0.001
PREC (L)–PHC (R)	−3.45	0.001
aHIPPO (R)–ANG (L)	−3.37	0.001
PREC (L)–AMYG (L)	−3.35	0.001

PREC, precuneus; AMYG, amygdala; pHIPPO, posterior hippocampus; aHIPPO, anterior hippocampus; PHC, parahippocampal cortex; OFC, orbitofrontal cortex; PRC, perirhinal cortex; RSC, retrosplenial cortex; PCC, posterior cingulate cortex; ANG, angular gyrus; L, left; R, right; SCH, schizophrenia group; HC, healthy control group; FC, functional connectivity; ROI, region of interest.

#### 3.2.2. Group differences in FC between networks

**Figure 3:**
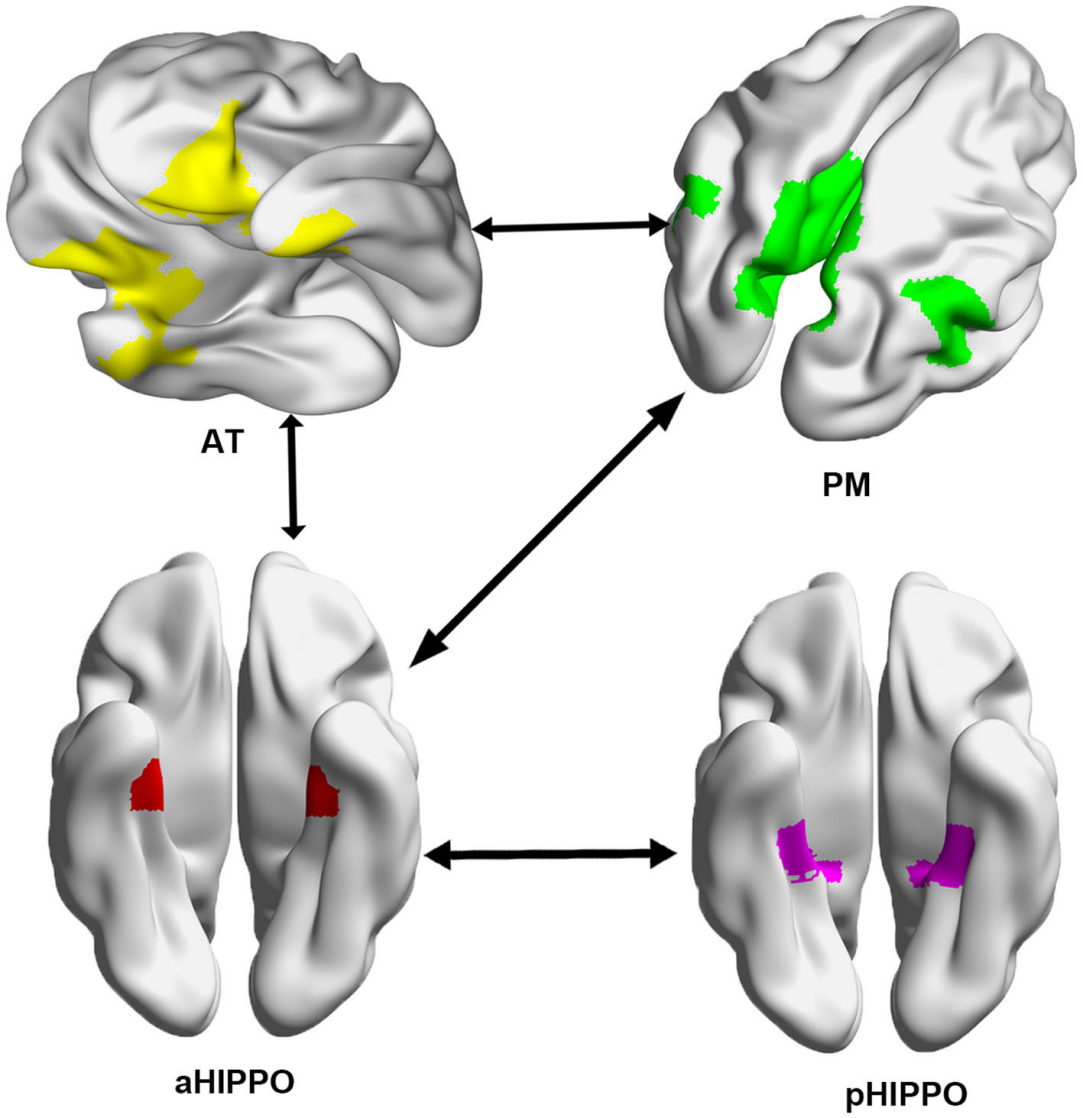
Network connections showing a significant decrease in large-scale between-network FC in the SCH group compared with the HC group. Arrows between systems indicate a decrease in FC between networks. L, left; R, right; PM, posterior medial system; AT, anterior temporal system; aHIPPO, anterior hippocampus; pHIPPO, posterior hippocampus.

**Figure 4:**
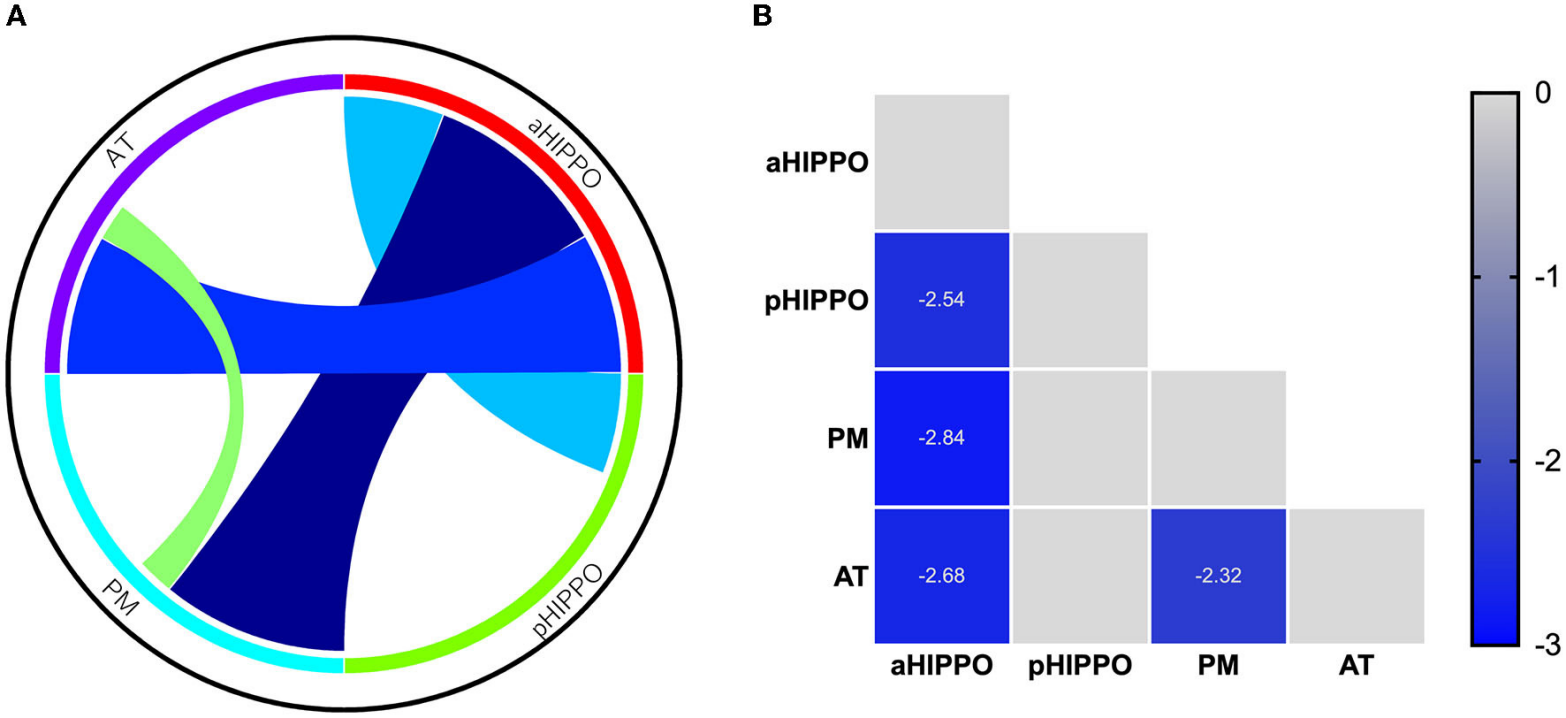
Results of analyses of between-network functional connectivity. **(A)** Differences in between-network functional connectivity between SCH patients and the HC group. **(B)** Between-network functional connectivity matrix. Pairwise correlations between cortico–hippocampal networks were averaged across subjects. Cooler colors represent decreased between-network FC in schizophrenia patients compared to healthy controls. PM, posterior medial system; AT, anterior temporal system; aHIPPO, anterior hippocampus; pHIPPO, posterior hippocampus.

Compared to the HC group, SCH patients showed four significantly different edges in the connections between cortico–hippocampal networks, representing significantly decreased FC between the AT and the PM; the AT and the aHIPPO; the PM and the aHIPPO; and the aHIPPO and the pHIPPO (Figures 3, 4, Table 4).

**Table 4:** Differences in large-scale between-network FC.

**Between-network connections (SCH > HC)**	***t***	***P***
aHIPPO–PM	−2.84	0.005
aHIPPO–AT	−2.68	0.008
aHIPPO–pHIPPO	−2.54	0.012
AT–PM	−2.32	0.021

aHIPPO, anterior hippocampus; PM, posterior medial system; AT, anterior temporal system; pHIPPO, posterior hippocampus; SCH, schizophrenia group; HC, healthy control group; FC, functional connectivity.

### 3.3. Spearman correlational analysis

**Figure 5:**
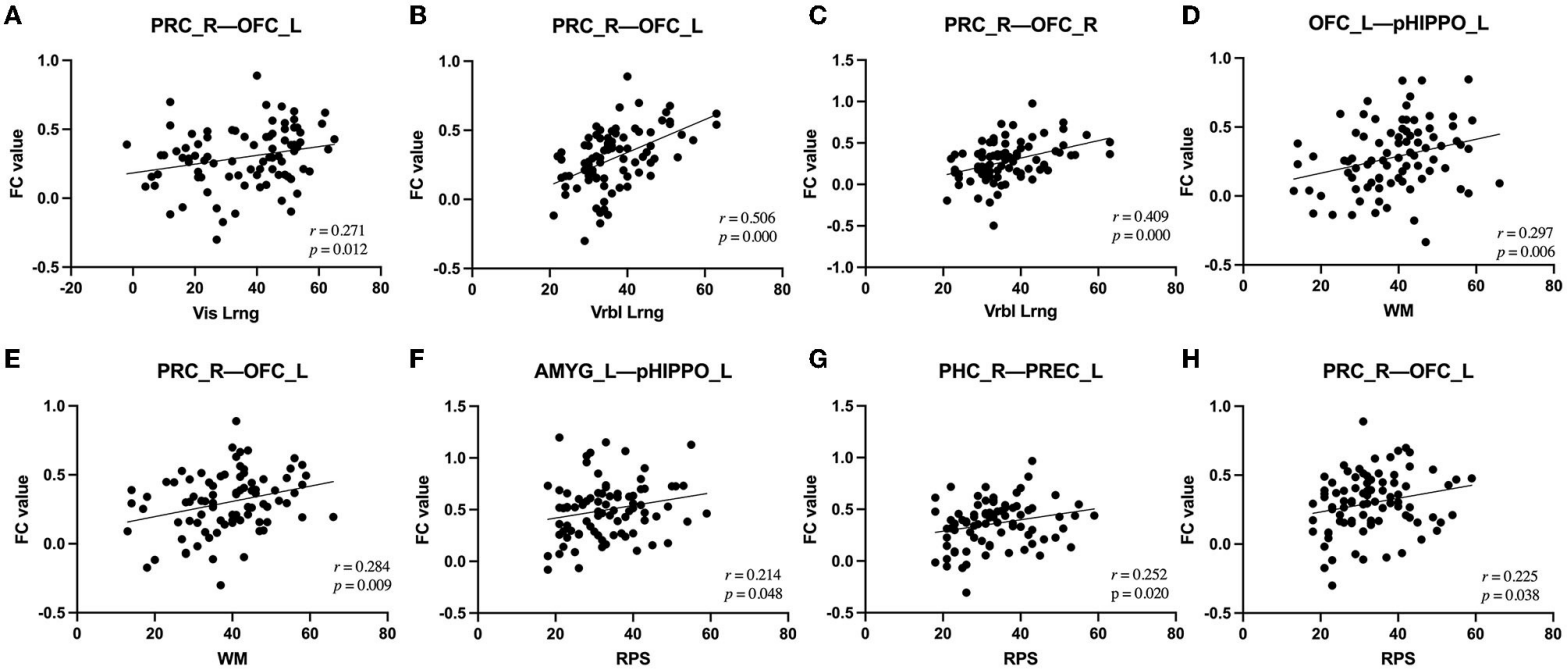
Correlations between alterations in FC and clinical outcomes in patients with SCH. **(A)** Alteration of right PRC–left OFC FC was positively correlated with change in Vis_Lrng score (*p* = 0.012, *r* = 0.271). **(B)** Alteration of right PRC–left OFC FC was positively correlated with change in Vrbl_Lrng score (*p* = 0.000, *r* = 0.506). **(C)** Alteration of right PRC–right OFC FC was positively correlated with change in Vrbl_Lrng score (*p* = 0.000, *r* = 0.409). **(D)** Alteration of left OFC–left pHIPPO FC was positively correlated with change in WM score (*p* = 0.006, *r* = 0.297). **(E)** Alteration of right PRC–left OFC FC was positively correlated with change in WM score (*p* = 0.009, *r* = 0.284). **(F)** Alteration of left AMYG–left pHIPPO FC was positively correlated with change in RPS score (*p* = 0.048, *r* = 0.214). **(G)** Alteration of right PHC–left PREC FC was positively correlated with change in RPS score (*p* = 0.020, *r* = 0.252). **(H)** Alteration of right PRC–left OFC FC was positively correlated with change in RPS score (*p* = 0.038, *r* = 0.225). FC, functional connectivity; PREC, precuneus; AMYG, amygdala; pHIPPO, posterior hippocampus; aHIPPO, anterior hippocampus; PHC, parahippocampal cortex; OFC, orbitofrontal cortex; PRC, perirhinal cortex; L, left; R, right; ROI, region of interest; WM, working memory; Vrbl_Lrng, verbal learning and memory; Vis_Lrng, visual learning and memory; RPS, reasoning and problem-solving.

Among the fifteen instances of significantly different within-network FC and the four instances of significantly different between-network FC, after controlling for the confounders of sex, age, education, and mean FD, there were various correlations with cognitive and PANSS scores. In terms of within-network FC, PHC (R)–PREC (L) FC was significantly negatively correlated with AV score and significantly positively correlated with Vis_Lrng score; PRC (R)–OFC (L), PRC (R)–OFC (R), and OFC (L)–pHIPPO (L) FC were significantly positively correlated with Verbl_Lrng score; and OFC (L)–pHIPPO (L) and PRC (R)–OFC (L) FC were significantly positively correlated with WM score. Additionally, within-network AMYG (L)–pHIPPO (L), PHC (R)–PREC (L), and PRC (R)–OFC (L) FC and between-network aHIPPO–pHIPPO FC were significantly positively correlated with WM score; within-network ANG (L)–aHIPPO (R), PHC (R)–PREC (L), PHC (R)–PREC (R), PREC (L)–aHIPPO (R), and PREC (R)–aHIPPO (R) FC and between-network aHIPPO–pHIPPO and aHIPPO–PM FC were significantly positively correlated with SC score; within-network PREC (R)–aHIPPO (R) FC was significantly negatively correlated with positive PANSS score; within-network AMYG (L)–pHIPPO (L) FC and between-network aHIPPO-AT FC were significantly negatively correlated with negative PANSS score; and within-network PHC (R)–PREC (L), PHC (R)–PREC (R), and PHC (R)–PREC (R) FC were significantly positively correlated with total PANSS score (Figure 5, Table 5).

**Table 5:** Correlational analysis between altered FCs with PANSS and cognitive scores.

**Clinical variable**	ROI–ROI/ network–network (^*^) connection	***r***	***P***
PANSS_P	PREC (R)–aHIPPO (R)	−0.249	0.021
PANSS_N	aHIPPO–AT^*^	−0.298	0.005
	AMYG (L)–pHIPPO (L)	−0.231	0.033
PANSS_T	PHC (R)–PREC (L)	0.229	0.034
	PHC (R)–PREC (R)	0.223	0.039
AV	PHC (R)–PREC (R)	−0.227	0.036
Vis_Lrng	PRC (R)–OFC (L)	0.271	0.012
Verbl_Lrng	PRC (R)–OFC (L)	0.506	0.000
	PRC (R)–OFC (R)	0.409	0.000
WM	OFC (L)–pHIPPO (L)	0.297	0.006
	PRC (R)–OFC (L)	0.284	0.009
RPS	aHIPPO–pHIPPO^*^	0.236	0.029
	AMYG (L)–pHIPPO (L)	0.214	0.048
	PHC (R)–PREC (L)	0.252	0.020
	PRC (R)–OFC (L)	0.225	0.038
SC	aHIPPO–pHIPPO^*^	−0.226	0.037
	aHIPPO–PM^*^	−0.315	0.003
	ANG (L)–aHIPPO (R)	−0.299	0.005
	PHC (R)–PREC (L)	−0.279	0.009
	PHC (R)–PREC (R)	−0.219	0.043
	PREC (L)–aHIPPO (R)	−0.313	0.003
	PREC (R)–aHIPPO (R)	−0.293	0.006

PREC, precuneus; AMYG, amygdala; pHIPPO, posterior hippocampus; aHIPPO, anterior hippocampus; PHC, parahippocampal cortex; OFC, orbitofrontal cortex; PRC, perirhinal cortex; RSC, retrosplenial cortex; PCC, posterior cingulate cortex; ANG, angular gyrus; L, left; R, right; ROI, region of interest; PM, posterior medial system; AT, anterior temporal system; PANSS_P, positive PANSS score; PANSS_N, negative PANSS score; PANSS_T, total PANSS score; SOP, speed of processing; AV, attention/vigilance; WM, working memory; Vrbl_Lrng, verbal learning and memory; Vis_Lrng, visual learning and memory; RPS, reasoning and problem-solving; SC, social cognition.

* represent between network FC alterations.

## 4. Discussion

Through comparison of large-scale rs-FC analysis of the cortico–hippocampal network between first-episode schizophrenia patients and healthy controls, our study provided a description of the modular integration and separation patterns of the AT system, the PM system, the anterior hippocampus, and the posterior hippocampus. Our results indicated that FC within and between the cortico–hippocampal network was significantly decreased in first-episode schizophrenia patients compared with the healthy control group, affecting 24 brain regions; this strongly validates the hypothesis of cortico–hippocampal dysconnectivity. Furthermore, this cortico–hippocampal dysconnectivity was significantly related to the severity of symptoms and cognition deficits in schizophrenia, suggesting its pathophysiological relevance. FC values for several areas of decreased FC were positively correlated with Vis_Lrng, Verbl Lrn, WM, RPS, and total PANSS scores and negatively correlated with AV, SC, positive PANSS, and negative PANSS scores. These findings provide new insights into the potential neurobiology of schizophrenia from the perspective of network modularity, as we discuss briefly below.

### 4.1. Within-network FC abnormalities in SCH

At the within-network level, we discovered decreased intrinsic FC in the cortico–hippocampal network, including the AT system, the PM system, and the anterior and posterior hippocampus, which is partially consistent with prior reports for schizophrenia (Benetti et al., 2009; Belujon et al., 2013; Liu et al., 2020; Wang et al., 2021; Ikeda et al., 2022; Walther et al., 2022). The cortico–hippocampal network plays a critical role in cognition, especially memory: AT is mainly involved in recognition and associative memory, affective processing, semantic processing, and object perception (Diana et al., 2007), while PM is implicated in episodic and autobiographical memory for the context of an event, space and time, scene perception, and social cognition (Johnson and Rugg, 2007). We identified altered FC for 15 ROI–ROI connections within the cortico–hippocampal network, with twelve involving the AT system, seven involving the PM system, four involving the anterior hippocampus, and three involving the posterior hippocampus. In addition, four instances of altered FC for ROI–ROI connections were observed within the AT system, but none occurred within the PM system. Therefore, in summary, the AT system exhibited more FC alterations than the PM system in schizophrenia patients, both within the AT system and between AT and other systems, suggesting that the AT system may play a more significant role than the PM system in the pathogenesis of schizophrenia.

Our study found that the precuneus was the brain region that showed altered FC with the greatest number of other brain regions, including the amygdala, PHC, and anterior hippocampus; the most significant decrease in FC was observed between the precuneus and the amygdala. The precuneus is a critical node in the default mode network (DMN), which has been found in numerous studies to be closely related to reflective and self-related processing, awareness and conscious information processing, empathy, episodic memory, and visuospatial processing (Dörfel et al., 2009; Harvey et al., 2013). There is growing evidence that the precuneus plays a significant role in self-processing, insight, and empathy deficits in schizophrenia (Faget-Agius et al., 2012). Multiple alterations to FC of the precuneus with other nodes in the DMN have been reported in schizophrenia patients (Hu et al., 2017), as well as altered resting state and task-related activity/deactivation of the precuneus (Garrity et al., 2007; Kühn and Gallinat, 2013). Zhang et al. (2020) also identified decreased network homogeneity of the posterior cingulate cortex/precuneus in the DMN, which is correlated with neurocognitive deficits in drug-naive first-episode adolescent-onset schizophrenia. All these studies indicate that dysfunction of FC in the DMN plays a key role in cognitive impairment in schizophrenia, especially in executive function and memory. Gong et al. (2014) found that the precuneus is closely associated with DISC1 polymorphisms and negative symptom severity in schizophrenia. Dörfel et al. (2009) observed that the connectivity between the precuneus and the hippocampus is involved in the recognition memory network. The hippocampus, PHC, and amygdala are critical constituents of the cortical and subcortical limbic system and participate in the regulation of emotional reactions, learning, memory, and behavior. In this study, the functional connectivity of the precuneus with the amygdala, hippocampus, and PHC is weakened, which is consistent with the findings of a study by Wen et al. (2021), suggesting that the altered connectivity between the precuneus and the limbic system lays the neurobiological foundation for the decline of cognitive function and intensification of negative symptoms in schizophrenia (Berman et al., 2016; Rebouças et al., 2018). Our study also identified decreased FC between the RSC and the PCCR. The RSC and PCC are key components of the core composition of the DMN and are collectively referred to as the posterior DMN (pDMN) and posterior cingulate gyrus. Mounting evidence has indicated the functional segregation of the PCC and the RSC (Rolls et al., 2022a). Kaboodvand et al. (2018) reported that the RSC is a crucial gateway to episodic memory through its connections with subcortical and cortical subsystems of the DMN, including the PCC. Reduced FC between the OFC and the PRC is another considerable distinction between schizophrenia patients and healthy controls. Weaker effective connectivity was found in the path from the OFC to the entorhinal, perirhinal, and parahippocampal cortex in a study by Rolls et al. (2022b); this region is involved in memory and navigation in humans.

In addition to these findings, we also observed multiple instances of dysconnectivity between the hippocampus and other regions within the cortico–hippocampal network, including the amygdala, PREC, OFC, and ANG. As a crucial hub component of the limbic system, the hippocampus has been found to support memory and other behaviors, including learning and integration of information (Xiu et al., 2021), and is essential for encoding and retrieval of the context of personal events (Tulving and Markowitsch, 1998). Schizophrenia has been proven to be closely associated with structural and functional impairment of the hippocampus (Xiu et al., 2021), which is regarded as an important biomarker in the pathophysiology of schizophrenia and is involved in early detection and intervention (Lieberman et al., 2018). The amygdala has been proven to be related to recognition of negative facial emotions and threats, and to the formation of credibility judgments; this region is a central hub in the social brain network, and impairment of the amygdala has been found to affect social judgment in patients with schizophrenia (Mukherjee et al., 2014). Previous studies have found that aberrant patterns of functional connectivity in the amygdala and hippocampal neural loop of the cortical-limbic system are observed in schizophrenia patients (Comte et al., 2018; Wang et al., 2021). The AG is the core hub in multiple subsystems of the brain network, affecting memory, semantic processing, reading, and word comprehension, and has been reported on frequently in the recent literature on episodic memory (Benoit and Schacter, 2015; Bellana et al., 2016). Several studies have discovered that asymmetric deterioration of the AG and a decrease in the amplitude of low-frequency fluctuations are associated with auditory hallucinations and confusion in schizophrenia patients (Niznikiewicz et al., 2000; Gao et al., 2022). Given the role of the angular gyrus and the hippocampus in episodic memory, the weakening of the FC between the angular gyrus and hippocampus observed in this study may represent the neurobiological mechanism of episodic memory deficits in schizophrenia.

### 4.2. Between-network FC abnormalities in SCH

In the interaction of multiple networks, establishment of the most suitable equilibrium between synchronization within the network and coupling between networks is crucial for high-level emotional and cognitive processes (Berman et al., 2016). There have been several studies describing alterations in FC networks in cognitively impaired schizophrenia patients, the majority of which have focused on whole-brain networks or the DMN (Zhang et al., 2020); few studies have focused their attention on the AT and PM networks, which are intensively interconnected with the MTL. At the between-network level, our study observed decreased FC between the aHIPPO and PM, the aHIPPO and AT, the aHIPPO and pHIPPO, and the AT and PM. Altered FC between the pHIPPO and PM or AT was not observed in this study.

The PM system has been found to be linked to episodic and autobiographical memory, space and time, scene perception, and social cognition, while the AT system is closely associated with recognition and associative memory, affective processing, semantic processing, and object perception. The PM and AT systems must work collaboratively to support integral aspects of cognitive behavior, including memory, which involves connections via a crucial hub, the hippocampus (Barnett et al., 2021). Functional dissociation along the longitudinal axis of the hippocampus may explain the distinction in functional connectivity between the anterior and posterior hippocampus with the PM and AT systems (Dugré et al., 2021). Converging evidence from multiple studies indicates that the anterior hippocampus is involved in the first-episode psychosis stages of schizophrenia, with structural and functional alterations (McHugo et al., 2018; Blessing et al., 2020); this is consistent with the multiple instances of functional dysconnectivity of the anterior hippocampus with other systems observed in our study, with the focus in this study being on first-episode schizophrenia patients. This indicates that the anterior hippocampus may mediate the AT and PM systems, aberrant connectivity of which may trigger the onset of symptoms and cognitive deficits in first-episode schizophrenia. Interestingly, a significant decline of functional connectivity between the anterior and posterior hippocampus was also observed in our study. Few previous studies have examined functional connectivity within the hippocampus, and this additional result may reveal the critical role of alterations to functional integration and connectivity in the hippocampus in bringing about impairments to memory and other cognitive functions.

### 4.3. Correlations between within- and between-network FC and behavior

The correlations observed between aberrant within- and between-network FC and the degree of cognitive deficits in schizophrenia indicate that FC decreases as cognition becomes increasingly impaired, with scores on working memory, verbal learning and memory, visual learning and memory, reasoning, and problem-solving showing positive correlations with the reduction in FC. It has been reported previously that the PMAT system has close involvement with multiple facets of cognitive processing (Ritchey et al., 2015). Although the PM and AT systems cooperate in functions relating to the item and context information, emotional and semantic information, and other event-related details, the functional separation of PM and AT in the resting state is of increasingly concern (Cooper and Ritchey, 2019). Numerous studies have shown that schizophrenia patients experience extensive cognitive impairment. Measurement of intrinsic fluctuations in the neural activity that is considered to support cognition, through the resting-state fMRI method, is essential to understand the neural correlates of cognitive impairment in schizophrenia. Our findings of correlations of decreased FC among the AT, PM, and hippocampus with cognition may be consistent with those of Adhikari et al. (2019), who observed extensively impaired rs-FC in the salience, sensorimotor, auditory, default mode, and other functional networks that are thought to support cognitive function in terms of both within- and between-network functional connectivity in schizophrenia patients, in consideration of the preliminary foundation that multiple anomalies of between-network connectivity are expected to become representative of the network phenotypes of psychiatric disorders (Seeley et al., 2007; Li et al., 2019; Rodriguez et al., 2019). Our findings emphasize the significant role of decreased cortico–hippocampal network functional connectivity in cognitive deficits in schizophrenia, which may represent a new avenue for understanding of the specialized neural representation of different domains of cognitive function. In our study, there were 22 instances of altered ROI–ROI and network–network FC that were significantly correlated with PANSS and cognitive scores, among which 17 involved the AT system, especially affecting the cognitive functions of Vis_Lrng, Verb_Lrng, WM, and RPS, which were positively associated with the degree of altered FC. These striking results re-emphasize the fundamental contribution of the AT system in the pathogenesis of schizophrenia patients, which is especially strongly associated with the cognitive domains of memory, reasoning, and problem-solving.

### 4.4. Limitations

There are several limitations to this study that need to be addressed. First, this was a cross-sectional study of pre-medication schizophrenia patients. Therefore, it is important to replicate the findings of alterations in the specific functional connectivity of the cortico–hippocampal system using longitudinal fMRI data to determine whether a given patient will progress between different states. Second, our study utilized resting-state functional magnetic resonance data, and it is difficult to determine aberrant patterns of functional connectivity in the hippocampal-AT/PM system when patients are suffering from related symptoms or performing diverse cognitive tasks. Hence, conducting fMRI scanning during concurrent performance of distinct cognitive tasks by the subjects can help to judge the cortico–hippocampal network connectivity state corresponding to the relevant cognitive functions. Finally, while the study was not particularly small, making strong, reproducible claims about these results (correlations between RSFC and behavioral phenotypes such as cognitive test performance or positive/negative symptoms) would require thousands of individuals (Marek et al., 2022), and our results should be followed up with a larger sample in future work.

## 5. Conclusion

Taking the findings together, this study detected, using the resting-state fMRI method and large-scale edge-based network analysis, decreased within-network and between-network FC of the cortical-hippocampus network involving the AT, PM, aHIPPO, and pHIPPO systems (especially the AT system and the aHIPPO) and associated with cognitive impairment (mainly in the domains of Vis_Lrng, Verb_Lrng, WM, and RPS). The large-scale brain network results demonstrated that the integration and separation of AT/PM-aHIPPO/pHIPPO in terms of functional connectivity could serve as cognition-specific neurofunctional markers in schizophrenia (especially for memory), supplying several new insights into the neurobiology of schizophrenia.

## Data availability statement

The original contributions presented in the study are included in the article/supplementary material, further inquiries can be directed to the corresponding author.

## Ethics statement

The studies involving human participants were reviewed and approved by the Ethics Committee of the First Affiliated Hospital of Zhengzhou University. Written informed consent to participate in this study was provided by the participants' legal guardian/next of kin.

## Author contributions

KX contributed to the conception and design of this study and wrote the first draft of the manuscript. JChen and YW recruited the participants and performed the MRI examinations. KX, SH, and CW processed the data. YC performed the statistical analysis. YZ, XS, and JCheng provided critical revisions to the manuscript. All authors contributed to the article and approved the submitted manuscript.
